# Editorial: World mental health day: mental health in the workplace

**DOI:** 10.3389/fpubh.2026.1844073

**Published:** 2026-05-15

**Authors:** Angela Stufano, Marília Silva Paulo

**Affiliations:** 1 Department of Surgical and Medical Science, University of Foggia, Foggia, Italy; 2 NOVA National School of Public Health, Public Health Research Center, Comprehensive Health Research Center, CHRC, CCAL, REAL, Universidade Nova de Lisboa, Lisboa, Portugal

**Keywords:** mental health in the workplace, occupational burnout, work environment, workplace wellbeing, world mental health day

The global observance of World Mental Health Day provides a timely opportunity to reflect on the central role of mental health in shaping the sustainability of modern work environments and the wellbeing of the workforce. Across sectors and professional roles, growing evidence indicates that mental health in the workplace is influenced by a complex interplay of organizational demands, interpersonal relationships, and individual coping capacities. Contemporary work settings are increasingly characterized by high workloads, evolving technological demands, and intensified performance expectations, all of which contribute to heightened psychological strain and vulnerability to mental health risk factors and/or even the development of burnout. At the same time, workplaces also represent critical environments for prevention, early detection, and the promotion of psychological resilience through supportive leadership, healthy organizational cultures, and access to appropriate resources.

This Research Topic aimed to produce comprehensive insights into the state of mental health in workplaces globally and identify effective strategies to support employees' mental wellbeing. From the 68 studies submitted, 40 were published: one systematic review, one brief research support and 38 original research papers. Half of the studies were from Asia (23% from China, Indonesia, and South Korea) but the Research Topic also represents European countries (Italy, Portugal, Austria, the United Kingdom, and Slovakia), North American (the United States of America), and Oceania (New Zealand). Collectively these body of literature underscore the need to view mental health as a shared organizational and societal responsibility, requiring coordinated, system-level strategies to foster safe, supportive, and sustainable working conditions across diverse occupational contexts.

The most studied occupational context was the healthcare sector with 20 studies, where 12 of them were on nurses. Figure 1 presents the treemap of the occupational setting studied in the Research Topic.

**Figure 1:**
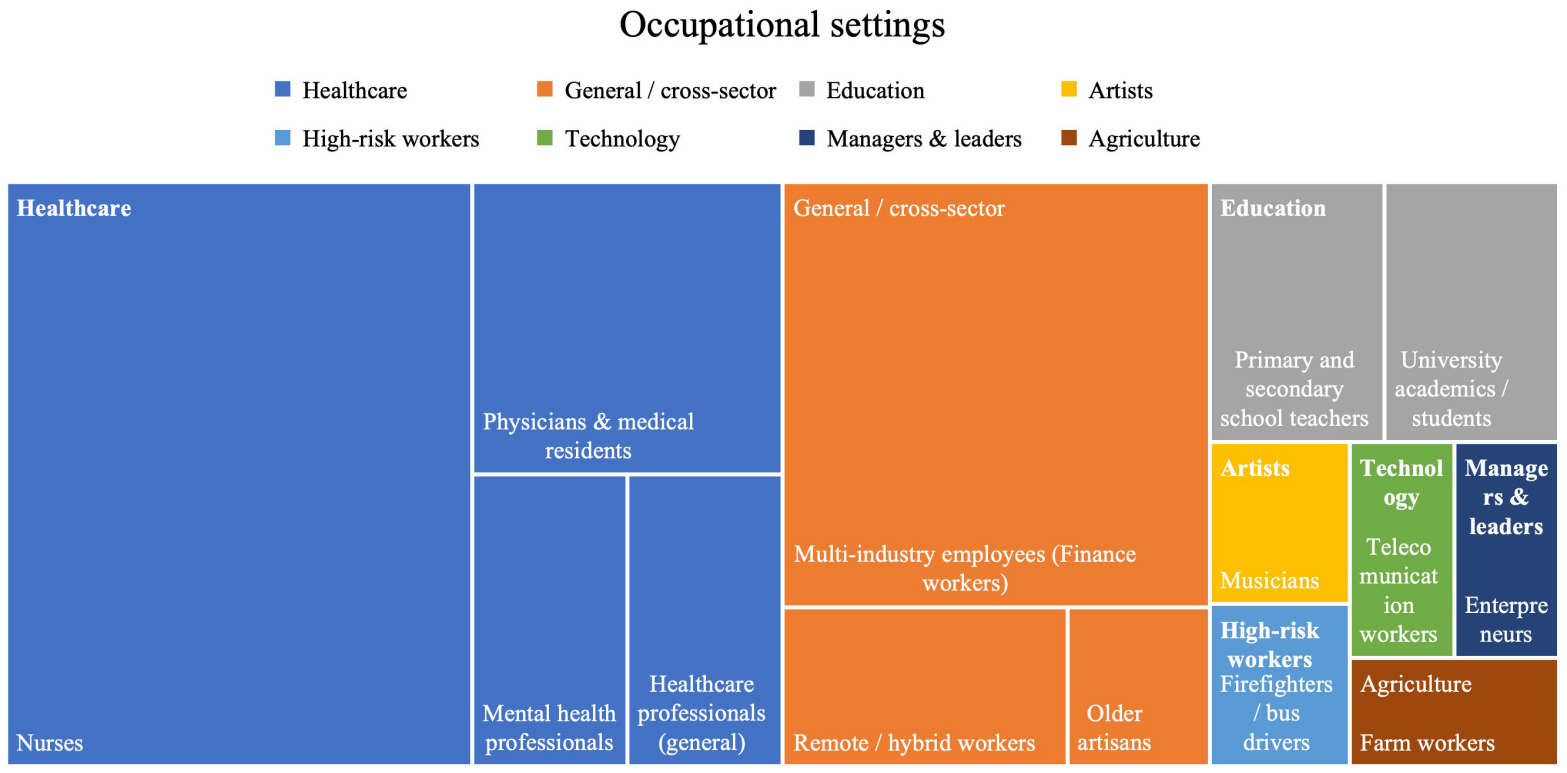
Treemap of the occupational settings represented in the Research Topic and studied for mental health conditions.

## 1 Workplace violence and interpersonal conflict as systemic occupational stressors

One of the most consistent and cross-cutting themes emerging from the literature relates to the impact of workplace violence and interpersonal conflict on the mental health of healthcare professionals, regardless of professional role or clinical setting. In both nursing and medical contexts, exposure to unsafe work environments is repeatedly associated with increased emotional distress, occupational stress, and reduced professional wellbeing. Zhou and Zeng demonstrated that nurses exposed to frequent and diverse forms of workplace violence reported significantly higher levels of burnout, emotional strain, and reduced perceived organizational support, highlighting the cumulative psychological burden associated with unsafe work environments. Similarly, Long et al. identified a direct association between exposure to violence and increased occupational stress among emergency department nurses, reinforcing the role of workplace safety as a fundamental determinant of psychological wellbeing. Evidence from broader healthcare populations further confirms that workplace violence represents a pervasive threat to mental health across professional groups. Robles et al. showed that occupational violence among mental healthcare professionals remains a widespread concern and is strongly associated with emotional distress, particularly among younger staff and those working in high-risk clinical environments. Consistent with this pattern, Vitale et al. demonstrated that exposure to workplace violence is linked to long-term psychological distress and diminished professional wellbeing, underscoring the enduring psychological consequences of unsafe clinical conditions. Together, these findings suggest that workplace violence should be understood not as an isolated incident but as a structural occupational hazard with sustained implications for workforce stability.

Beyond explicit violence, interpersonal conflict, and relational strain within healthcare teams also emerge as critical psychosocial risk factors. Using network analysis, Qiu et al. identified workplace conflict as a central driver of emotional exhaustion and psychological distress among mental health nurses, suggesting that strained professional relationships can amplify existing occupational stressors. In complex healthcare environments characterized by time pressure, hierarchical structures, and high emotional demands, unresolved interpersonal tensions may accumulate over time, contributing to chronic stress and reduced psychological resilience. These relational dynamics highlight the importance of organizational climate and team cohesion as protective factors in maintaining mental health.

## 2 Organizational pressures, workload, and work–life imbalance as structural determinants of mental health

A second major theme emerging in healthcare populations in general, and specifically in nurses, concerns the structural pressures associated with workload intensity, staffing constraints, and the challenge of maintaining a sustainable balance between professional and personal responsibilities. Across clinical settings, excessive workload, long working hours, and insufficient organizational support consistently emerge as central determinants of psychological strain and workforce instability. Hu et al. demonstrated that work–family conflict represents a persistent predictor of turnover intention among nurses across generational cohorts, underscoring the role of role conflict as a key driver of workforce attrition. Complementing this perspective, Zeng et al. showed that work–family conflict, particularly when combined with maladaptive coping strategies, significantly increases occupational fatigue, highlighting the cumulative impact of competing professional and family demands on both physical and mental health.

Evidence from physician and multidisciplinary healthcare contexts further reinforces the structural nature of these risks. Dholakia et al. identified workload intensity and lack of institutional resources as major contributors to burnout among surgical professionals, while Simões et al. demonstrated through a Delphi study that system-level factors—such as staff shortages and unfavorable working conditions—represent the most significant determinants of physician burnout within national health systems. Similarly, Zhang C. Q. et al., found that long working hours and heavy workloads remain central predictors of burnout among intensive care unit healthcare workers, even in the post-pandemic period. Importantly, their findings also indicate that access to workplace mental health resources significantly improves preventive behaviors and risk awareness, emphasizing the protective role of organizational support structures.

## 3 Emotional exhaustion and the psychological demands of clinical responsibility

The demanding nature of nursing work, particularly in high-risk clinical settings, such as operating rooms and emergency departments, emerged as another central source of psychological strain. A qualitative exploration of operating room nurses' experiences revealed how systemic pressures, high levels of responsibility, and constant exposure to critical clinical situations contribute to emotional exhaustion and professional fatigue (Li, Xiang et al.). These findings align with evidence from Wu et al., who showed that moral distress significantly contributes to burnout among emergency nurses, particularly in contexts where organizational support and ethical leadership are perceived as insufficient.

Together, these studies highlight the psychological consequences of sustained responsibility and ethical tension in clinical practice. Nurses frequently operate in environments where high-stakes decision-making, time pressure, and moral dilemmas are routine, creating conditions that can erode emotional resilience and increase susceptibility to burnout.

## 4 Work–life balance, social relationships, and occupational integration

Beyond workload and organizational demands, several studies emphasize the critical role of work–life balance and interpersonal relationships in shaping mental health outcomes. Li N. et al. demonstrated that work–family conflict is strongly associated with workplace ostracism among healthcare workers, particularly when individuals lack sufficient psychological resources to manage competing role demands. Similarly, Mincarone et al. showed that worsening depressive symptoms and deteriorating interpersonal relationships significantly influence workers' perceptions of their professional experience, particularly in remote or hybrid work arrangements after the COVID-19 pandemic.

These findings indicate that social integration and relational stability are essential components of occupational wellbeing. In healthcare environments characterized by high responsibility and emotional demands, the inability to maintain supportive social relationships, both inside and outside the workplace, can amplify psychological stress and increase vulnerability to burnout.

## 5 Measurement and adaptive tools for workplace recognition of burnout as a systemic occupational health priority

An additional cross-cutting theme emerging from the literature concerns the critical role of reliable assessment tools in identifying, monitoring, and preventing burnout across healthcare professions. The availability of valid and context-specific measurement instruments is essential for recognizing early signs of psychological distress, particularly in high-intensity clinical environments where emotional exhaustion may develop gradually and remain unreported. In this context, measurement should be understood not merely as a technical task but as a foundational component of occupational health protection and workforce sustainability.

Evidence from specialized clinical settings illustrates the importance of culturally and professionally tailored assessment tools. One study validated the Chinese version of the Occupational Burnout Scale for operating room nurses, confirming its reliability and relevance for high-pressure surgical environments characterized by sustained responsibility and emotional strain (Li, Liu et al.). This methodological advancement supports more accurate identification of psychological risk and enables targeted organizational interventions to address emerging mental health needs. By improving the precision of burnout assessment in specific clinical contexts, such tools contribute to earlier detection of distress and more effective prevention strategies.

At the system level, broader methodological challenges in burnout measurement remain evident. A systematic review of burnout assessment instruments used during the COVID-19 pandemic, identified substantial variability in the structure, reliability, and validity of existing tools, highlighting significant gaps in the standardization of occupational health measurement (Efremova et al.). The lack of consistent and validated instruments can delay the detection of psychological risk and reduce the effectiveness of preventive programs, particularly during periods of heightened organizational strain. These findings underscore the need for coordinated efforts to strengthen methodological rigor and harmonize measurement practices across healthcare systems.

## 6 Organizational leadership and psychological resources as integrated protective factors for mental health

While the studies published in this Research Topic consistently identify significant occupational risks across healthcare settings, they also converge on a set of protective factors that can mitigate psychological distress and sustain professional functioning. Leadership quality and organizational climate emerge as central determinants of workforce wellbeing, shaping both individual coping capacity and collective resilience. Liu, He, Tian, Guo et al. demonstrated that ethical leadership plays a critical role in improving nurses' job performance and wellbeing, partly through its influence on self-compassion, highlighting the importance of supportive management practices in promoting psychological stability. Similarly, Wu et al. identified hospital ethical climate and moral resilience as key factors in reducing burnout risk, emphasizing that organizational culture can function as a protective buffer against chronic occupational stress.

At the same time, the literature underscores the importance of individual psychological resources in sustaining engagement and adaptation in demanding clinical environments. Another study from Liu, He, Tian, Zhang et al. also showed that moral resilience mediates the relationship between self-compassion and work engagement among nurses, suggesting that internal coping capacities play a crucial role in maintaining motivation and performance under pressure. Consistent with this perspective, Peng et al. found that emotional intelligence and resilience significantly facilitate professional adjustment among novice nurses, particularly during early career transitions characterized by uncertainty and increased workload. These findings indicate that psychological competencies are essential tools for navigating complex professional roles and managing the emotional demands inherent in healthcare practice.

Evidence from broader healthcare populations further reinforces the role of adaptive personal capacities in shaping perceptions of work quality and professional sustainability. Zhang X. et al. demonstrated that self-control and career adaptability are positively associated with perceptions of decent work among psychiatrists, suggesting that flexible coping strategies and professional development capacities can enhance resilience in high-demand specialties. In addition, Chen et al. showed that gender differences influence how healthcare professionals experience and respond to occupational stress. For example, males were associated with having promotion pressure and females with medical dispute among them, emphasizing the need for context-sensitive interventions that account for individual characteristics and social roles. Together, these findings highlight the importance of tailoring occupational health strategies to the diverse needs of healthcare workers.

Additional evidence highlights the role of leadership and organizational communication in shaping wellbeing. Koša and Lisá examined how perceptions of meaningful work and workplace attachment styles predict wellbeing among entrepreneurs operating within structured sales networks. Their findings demonstrated that supportive leadership relationships, clear communication, and psychologically safe work environments were strongly associated with improved wellbeing, emphasizing the importance of relational trust and organizational climate in sustaining psychological health.

Taken together, the studies included in this Research Topic demonstrate that the interaction between organizational conditions, interpersonal relationships, and individual psychological resources shapes healthcare workers' wellbeing. Across diverse professional roles and healthcare clinical contexts, common risk factors include workload, violence exposure, and work–family conflict. These findings support the adoption of integrated, system-level strategies to promote workforce resilience and sustainable healthcare delivery.

## 7 Mental health and wellbeing in educational and academic settings

The challenges related to mental health and wellbeing extend beyond clinical environments into the educational sector, where teachers, educators, and students face unique systemic pressures. The transition from healthcare to education reveals parallel stressors, such as high emotional labor and work-life imbalance, yet introduces distinct professional dynamics.

Wang L. et al. explored the impact of work-family conflict on the occupational wellbeing of early childhood teachers through a complex chain mediation model. Utilizing a cross-sectional design, the study demonstrated that work-family conflict not only directly impairs wellbeing, but also operates through the sequential mediating roles of psychological empowerment and job crafting. Their findings suggest that when teachers feel empowered and are encouraged to proactively shape their work environment, the negative effects of domestic-professional interference are significantly mitigated. This emphasis on organizational support is echoed in the work of Xue et al., who investigated the sleep status and its influencing factors among primary and secondary school teachers in China. Their research highlighted a high prevalence of sleep disturbances linked to job strain and ruminative thoughts, identifying professional stress as a primary predictor of poor sleep hygiene, which subsequently deteriorates overall mental health.

In addition to traditional educational roles, the mental health of students, the future of the workforce, represents a critical area for intervention. Wei et al. contributed a methodological advancement by applying acoustic-based machine learning approaches for depression detection among university students. By analyzing vocal biomarkers through a cross-sectional study, they demonstrated that non-invasive, AI-driven tools can effectively identify early signs of depressive symptoms, offering a scalable solution for mental health screening in large academic populations.

The integration of wellness interventions within educational and community contexts requires participatory strategies to ensure sustainability and relevance. Frazier et al. provided a qualitative evaluation of a co-created wellness intervention for community health educators. Using a participatory approach to tailor the program to the specific context of the educators, the study underscored that “patience” and stakeholder engagement are essential for overcoming institutional barriers and fostering a culture of wellbeing. Furthermore, innovative workplace resources, such as “bring-your-dog-to-work” programs, were analyzed by Schieler et al. through a constant comparative analysis. Their results indicate that while such programs introduce specific demands, they serve as significant social and emotional resources, enhancing interpersonal connections and reducing the perceived stress associated with the academic and administrative workload. Collectively, these studies suggest that protecting mental health in the educational sector requires a dual focus on innovative detection technologies and the co-creation of supportive, flexible organizational cultures.

## 8 Working conditions and structural determinants of occupational mental health

Several contributions within this Research Topic address occupational mental health in heterogeneous working populations, underscoring the transversal nature of psychosocial risks across diverse professional contexts. Although these studies examine workers from different sectors—including telecommunications employees, young workers, mixed occupational groups, and employees in high-risk or high-demand environments—their findings converge on a shared observation: organizational demands, working conditions, and structural constraints play a central role in shaping psychological wellbeing and vulnerability to distress in contemporary workplaces.

A number of studies highlight how working conditions and organizational structures influence mental health outcomes. Wang and Zhang investigated the relationship between non-standard work schedules and self-rated mental health in a large national sample, demonstrating that irregular working hours were associated with poorer mental health through mechanisms such as increased work stress, work–family conflict, and reduced job satisfaction. Similarly, Zhang N. et al. examined occupational stress among workers in high-risk occupations, including firefighters, bus drivers, video display terminal operators, and port workers, and evaluated the effects of structured psychological interventions. Their results revealed substantial baseline levels of occupational stress and showed that sustained mental health training and group-based support were associated with meaningful reductions in stress over time, indicating the potential effectiveness of organizational prevention strategies.

Parallel evidence emerges from studies conducted in less traditional occupational settings. Humer et al. investigated mental health support needs among Austrian farmers, identifying a substantial subgroup experiencing elevated levels of psychological distress and expressing a clear demand for tailored support services. Their findings revealed that approximately one-third of participants reported a desire for mental health support, with common needs including counseling, practical assistance, and opportunities for rest and recovery, highlighting the importance of integrating psychosocial and structural interventions in rural occupational health strategies. At the same time, the study underscores the role of structural barriers, such as limited time, financial constraints, and geographic isolation, in restricting access to mental health services, thereby reinforcing the need for context-sensitive, accessible support systems in vulnerable occupational settings.

These body of evidence reinforces the centrality of structural and organizational conditions as foundational determinants of occupational mental health across diverse professional contexts.

## 9 Interpersonal dynamics and the social dynamics at work

Beyond structural conditions, several studies emphasize the importance of interpersonal relationships and social climate in shaping emotional wellbeing and psychological functioning in the workplace. These contributions demonstrate that relational dynamics can either amplify occupational stress or function as protective mechanisms that support mental health.

Cheng et al. explored the psychological consequences of perceived negative supervisor gossip, finding that employees exposed to such behaviors were more likely to engage in impression management strategies, which in turn increased emotional exhaustion. In a related context, Lee and Kim examined depressive symptoms among young workers (18–35 years old) and reported that workplace bullying was positively associated with depression, whereas job satisfaction functioned as a protective factor. Complementing these findings, Sheng et al. analyzed the role of work addiction among young employees and demonstrated that excessive work involvement was associated with reduced emotional intelligence and psychological detachment, ultimately contributing to greater interpersonal conflict.

Together, these studies suggest that relational strain, poor communication, and maladaptive social dynamics can significantly increase psychological vulnerability, even in occupational settings that are not traditionally considered high risk.

## 10 Psychological and social resources as protective factors

While structural and relational stressors represent significant risks, the literature also consistently highlights the role of psychological and social resources in mitigating occupational distress and supporting adaptive functioning. These resources operate at both the individual and contextual levels, enabling workers to regulate stress, maintain engagement, and sustain long-term wellbeing.

Song et al. investigated burnout among telecommunication employees and showed that family functioning was associated with lower levels of burnout both directly and indirectly through coping style and subjective wellbeing. These findings reinforce the view that psychological resilience is shaped not only by workplace conditions, but also by the broader social and family context.

Similarly, Sheng et al. identified emotional intelligence and psychological detachment as important mechanisms through which employees can regulate stress and maintain functional workplace relationships, highlighting the significance of recovery and emotional regulation in sustaining mental health.

Evidence from agricultural settings further underscores the importance of social support as a protective factor. Farmers experiencing limited social interaction and high workload demands may face increased risk of psychological distress, whereas strong social networks and accessible support services can facilitate coping and improve wellbeing (Humer et al.). These findings collectively demonstrate that resilience is not solely an individual attribute but a dynamic capacity shaped by social relationships, environmental conditions, and access to supportive resources.

## 11 Meaning, engagement, and identity in work

An emerging body of research within this Research Topic highlights the role of meaningful work, professional identity, and engagement as central determinants of occupational wellbeing. These studies suggest that psychological health is influenced not only by the absence of stressors but also by the presence of meaningful and socially valued work experiences.

Igreja et al. explored how older professional artisans perceive the impact of craftwork on their health and quality of life through a multi-methods approach combining standardized measures with in-depth qualitative interviews conducted in participants' workplaces. Their results indicate generally high levels of psychological wellbeing and life satisfaction among artisans, with participants frequently attributing their mental health to the meaningful, creative, and socially embedded nature of their work, while also identifying financial insecurity and physical workload as potential stressors. Importantly, the study highlights the generative role of creative work in promoting active aging and social participation, suggesting that engagement in culturally meaningful occupations can function as a protective factor for mental health across the life course.

A related line of inquiry emphasizes the role of subjective meaning and psychological attachment in entrepreneurial contexts. Koša and Lisá demonstrated that meaningful work is a robust predictor of psychological wellbeing, even when controlling for financial performance and organizational factors. These findings underscore the importance of aligning work activities with personal values and fostering a sense of purpose and belonging in professional life.

Taken together, these contributions reflect a growing recognition that occupational mental health is closely linked to the subjective experience of meaning, identity, and engagement in work.

## 12 Technological transformation and new work environments

Technological transformation and digitalization also emerge as critical themes in this body of research, reflecting the profound changes in work organization associated with digital technologies and remote work arrangements. These studies highlight both the opportunities and challenges created by technological innovation in modern workplaces.

Olivo et al. conducted a longitudinal study examining the transition to remote work within a public administration workforce, demonstrating that job autonomy and access to technological resources significantly enhance work engagement and improve employees' perceptions of their private lives. Their findings emphasize the importance of organizational policies that support digital adaptation and work–life balance in rapidly evolving work environments. At the same time, the study highlights the complex and context-dependent nature of remote work, showing that technological flexibility can generate both opportunities for wellbeing and new challenges related to social isolation and boundary management.

Innovative perspectives on digital work environments are further illustrated by Musgrave et al., who examined the role of social media in the mental health of professional musicians through a transdiagnostic cognitive-behavioral framework. Their qualitative findings reveal that social media functions simultaneously as a source of opportunity and risk, facilitating networking, creative expression, and professional visibility while also exposing workers to social comparison, online harassment, and performance pressures associated with the digital attention economy. This study contributes to a more nuanced understanding of digital labor by demonstrating that technological engagement is neither inherently beneficial nor harmful but depends on how individuals interact with digital platforms and manage their professional identities.

These findings collectively indicate that technological innovation is reshaping occupational environments in ways that require new approaches to mental health promotion and risk management.

## 13 Innovative approaches to assessment and intervention in occupational mental health

Several studies further contribute to the understanding of how occupational mental health can be monitored and improved through targeted interventions and innovative assessment approaches. These contributions reflect a growing emphasis on evidence-based strategies for preventing psychological distress and promoting wellbeing in diverse work environments.

Jingxuan et al. examined the association between psychological symptoms and HDL-related inflammatory indices, reporting significant relationships between these biological markers and levels of stress, anxiety, and depression by income, night work, and hours of work. Their findings suggest that objective physiological indicators may complement traditional self-report measures in identifying early signs of psychological distress.

In parallel, Shekhar et al., evaluated a workplace wellbeing training program for general workers in South Africa, using a mixed-methods design. They found that more intensive, in-person interventions were associated with improvements in wellbeing and work engagement, emphasizing the importance of structured, context-sensitive support initiatives.

Overall, these studies highlight the increasing relevance of multidisciplinary and data-driven approaches in occupational mental health research, demonstrating the value of integrating psychological, organizational, and biological perspectives in the design of effective prevention and intervention strategies.

